# Therapeutic Effect of Platelet-Rich Plasma Improves Bladder Overactivity in the Pathogenesis of Ketamine-Induced Ulcerative Cystitis in a Rat Model

**DOI:** 10.3390/ijms23105771

**Published:** 2022-05-21

**Authors:** Kuang-Shun Chueh, Kuan-Hua Huang, Jian-He Lu, Tai-Jui Juan, Shu-Mien Chuang, Rong-Jyh Lin, Yi-Chen Lee, Cheng-Yu Long, Mei-Chen Shen, Ting-Wei Sun, Yung-Shun Juan

**Affiliations:** 1 Graduate Institute of Clinical Medicine, College of Medicine, Kaohsiung Medical University, Kaohsiung 80708, Taiwan; spacejason69@yahoo.com.tw (K.-S.C.); urolong@yahoo.com.tw (C.-Y.L.); 2 Department of Urology, Kaohsiung Municipal Ta-Tung Hospital, Kaohsiung 80145, Taiwan; 3 Department of Urology, Kaohsiung Medical University Hospital, Kaohsiung 80708, Taiwan; u9181002@gmail.com (S.-M.C.); bear5824@gmail.com (M.-C.S.); selina750220@yahoo.com.tw (T.-W.S.); 4 Divisions of Urological Oncology, Department of Surgery, Chi Mei Medical Center, Tainan 71004, Taiwan; skhsteven@gmail.com; 5 Emerging Compounds Research Center, Department of Environmental Science and Engineering, College of Engineering, National Pingtung University of Science and Technology, Pingtung 91201, Taiwan; toddherpuma@yahoo.com.tw; 6 Department of Medicine, National Defense Medical College, Taipei 11490, Taiwan; terry870921@gmail.com; 7 Department of Parasitology, School of Medicine, College of Medicine, Kaohsiung Medical University, Kaohsiung 80708, Taiwan; rjlin@kmu.edu.tw; 8 Graduate Institute of Medicine, College of Medicine, Kaohsiung Medical University, Kaohsiung 80708, Taiwan; 9 Department of Medical Research, Kaohsiung Medical University Hospital, Kaohsiung 80708, Taiwan; 10 Department of Anatomy, School of Medicine, College of Medicine, Kaohsiung Medical University, Kaohsiung 80708, Taiwan; yichen83@kmu.edu.tw; 11 Department of Obstetrics and Gynecology, Kaohsiung Medical University Hospital, Kaohsiung 80708, Taiwan; 12 Department of Obstetrics and Gynecology, Kaohsiung Municipal Hsiao-Kang Hospital, Kaohsiung 80708, Taiwan; 13 Regenerative Medicine and Cell Therapy Research Center, Kaohsiung Medical University, Kaohsiung 80708, Taiwan

**Keywords:** bladder, platelet-rich plasma, ketamine, ulcerative cystitis, COX-2

## Abstract

The present study attempted to elucidate whether intravesical instillation of platelet-rich plasma (PRP) could decrease bladder inflammation and ameliorate bladder hyperactivity in ketamine ulcerative cystitis (KIC) rat model. Female Sprague Dawley (S-D) rats were randomly divided into control group, ketamine-treated group, ketamine with PRP treated group, and ketamine with platelet-poor plasma (PPP) treated group. Cystometry and micturition frequency/volume studies were performed to investigate bladder function. The morphological change of bladder was investigated by Mason’s trichrome staining. Western blotting analysis were carried out to examine the protein expressions of inflammation, urothelial differentiation, proliferation, urothelial barrier function, angiogenesis and neurogenesis related proteins. The results revealed that treatment with ketamine significantly deteriorated bladder capacity, decreased voiding function and enhanced bladder overactivity. These pathological damage and interstitial fibrosis may via NF-κB/COX-2 signaling pathways and muscarinic receptor overexpression. PRP treatment decreased inflammatory fibrotic biosynthesis, attenuated oxidative stress, promoted urothelial cell regeneration, and enhanced angiogenesis and neurogenesis, thereafter recovered bladder dysfunction and ameliorate the bladder hyperactivity in KIC rat model. These findings suggested that the PRP therapy may offer new treatment options for those clinical KIC patients.

## 1. Introduction

Ketamine is a noncompetitive N methyl-D-asparate receptor (NMDAR) antagonist and acting on glutamate binding sites, NMDA, and non-NMDA receptors. Clinically, ketamine abusers may develop severe lower urinary tract symptoms (LUTS), leading to increased urinary urgency, frequency, nocturia, dysuria, bladder pain and occasionally hematuria [1,2,3], similar to the symptoms of interstitial cystitis/bladder pain syndrome (IC/BPS) [1,2,3]. In addition, some ketamine abusers exhibit develops irreversible histological changes in the urinary tract. Reduced bladder compliance, low bladder capacity, detrusor overactivity, impaired renal function, and hydronephrosis have also been found in patients with ketamine-induced cystitis (KIC). Previous investigations suggested that toxic effects of ketamine metabolites result in mucosal ulceration, bladder barrier dysfunction, urothelial thinning, neurogenic inflammation, infiltration of granulocytes (mostly eosinophils) and mast cells, immunoglobulin E (IgE) mediated inflammation, and nitric oxide synthase mediated inflammation within bladder tissue, which related to the pathogenesis of KIC [4,5]. Moreover, KIC bladders were characterized by upregulation of transforming growth factor-β signaling-related genes, and phosphorylation of Smad2 and Smad3 proteins. In a mouse ketamine-addiction model, enhancement of non-cholinergic contractions and expression of P2X1 receptor in the bladder suggested that dysregulation of purinergic neurotransmission might cause overactivity of detrusor [6]. Ketamine can directly inhibit calcium influx and smooth muscle contractility by blocking L-type Ca^2+^ channel (Cav1.2), leading to voiding dysfunction [7]. Our previous study revealed that the up-regulation of cyclooxygenase-2 (COX-2) through the nuclear factor kappa B (NF-κB) pathway was involved in the inflammatory signaling of KIC in bladder urothelium. Promoter-deletion analysis showed that NF-κB was a necessary transcription factor for starting COX-2 gene [8].

Although several pathological changes had been investigated, the underlying pathophysiological mechanism of KIC related bladder dysfunction is still vague. Several potential mechanisms have been proposed, including (1) direct microvasculature toxicity leading to fibrosis and ischemia, (2) direct toxic effect of ketamine and its metabolite norketamine in the urine disrupting urothelial barrier and on mechanotransduction, which cause bladder hyperactivity, (3) autoimmune response resulting in bladder inflammation, and (4) neuronal toxicity induce nerve fiber degeneration that leads to voiding dysfunction [1,9,10,11].

Clinically, the therapeutic strategies for KIC patients include: (1) oral anti-muscarinic agents or/and ß3 agonist medication to decrease urinary frequency, nocturia and incontinence, (2) using analgesics to relieve bladder pain, (3) oral pentosan polysulfate for bladder mucosa repair, (4) antibiotic treatment to treat urinary tract infections (UTI) due to the fragile bladder mucosa, (5) intravesical hyaluronic acid (HA) instillation or botulinum toxin A (BoNT-A) injection, and (6) bladder hydro-distention under anesthesia or bladder mucosal cautery for bladder ulcer. However, more than 50% of patients discontinue the drug after half a year due to side effects. The BoNT-A injection not only blocked the presynaptic release of acetylcholine but also interfere with afferent nerve signaling. The side-effects of BoNT-A injection included acute urinary retention, large residual urine and hematuria. Therefore, the alternative method with high efficacy and safety for KIC patients is crucial.

Platelet-rich plasma (PRP) is a platelet-rich centrifugal autologous concentrate obtained by polymerization of platelet concentrates mixed with calcium and thrombin or aggregating factors [12,13]. Platelet could release cytokines and growth factors, included vascular endothelial growth factor (VEGF), transforming growth factor-β (TGF-β), epidermal growth factor (EGF), basic fibroblast growth factor (bFGF), insulin-like growth factor 1 (IGF-1) and platelet-derived growth factor (PDGF) [12,13,14,15,16]. These growth factors modulate tissue inflammation, increase tissue regeneration, promote tissue angiogenesis and increase blood flow in damaged tissue [12,13,17]. These molecules also regulate angiogenesis, remodel the extracellular matrix and influence the proliferation, differentiation, and recruitment of stem cells [16,18,19,20].

PRP has been designed into several kinds of product, such as platelet-rich fibrin, fibrin glue and platelet lysate. Platelet-rich fibrin is designed into solid or semi-solid platelet concentration membrane, which was used for soft tissue healing, bone graft protection and remodeling [13]. Fibrin glue is broadly used in different kinds of surgery for its adhesive and hemostatic action [13]. Perinelli et al. demonstrated the use and effect of fibrin glue on tendon rupture of a dog [21]. Platelet lysate was designed as the medium for cell culture instead of fetal bovine serum [13,16]. Platelet-rich plasma and fibrin, platelet lysate, and fibrin glue are widely used as nontransfusional blood components to improve the repair and regeneration processes after an injury [13]. Recently, PRP therapy has significantly expanded in veterinary medicine. Animal could be used as a study model for chronic and difficult-to-heal injuries mimicking human diseases [13].

The regenerative effects of PRP in a range of tissue types included skin, bone, cartilage, tendon and muscle, soft tissue, particularly in traumatic injury, orthopaedic, plastic surgery, poorly vascularised, chronic ulcers, and damaged tissue [12,16,22,23]. Previous studies have shown the use of PRP, adipose-derived mesenchymal stem cells (AD-MSCs), autologous fat grafting and biomaterials (HA and collagen) as new regenerative strategies for wound healing [22,23,24,25,26]. Moreover, PRP combined with AD-MSCs, autologous fat, or biomaterials treatment showed stronger regenerative potential in wound healing compared to AD-MSCs, autologous fat, or biomaterials alone [22,23,26]. In an in vitro study by Cervelli et al., they found that higher concentration of PRP could help adipose-derived stem cells maintain the fat volume of graft [26]. Clinically, PRP was also used to maintain the survival of fat graft [23,26]. The effect of PRP not only improved the speed of wound healing [13,20,27], but also reduced neurological and neuropathic pain associated with injuries [28,29]. For example, PRP were used to induce angiogenesis and regeneration of excisional wounds on mouse skin [13,20]. Clinically, PRP has been used as a therapeutic method for chronic mucostitis [30], diabetic foot and leg ulcers [31,32], chronic venous leg ulcer [33,34], chronic decubitus ulcers [35], tendon, muscle, bone, cartilage and wound healing [12,36,37,38]. A vivo study treating for tendon injury also showed PRP had a mitogenic effect on cells from endothelium, bone, cartilage and periodontal ligament [12]. Besides, increased angiogenesis and mitogenesis mediated by PRP obtained from diabetic patients were observed [20]. Moreover, the intravesical instillation of PRP increased the urothelial mitosis and improved hemorrhage in a cyclophosphamide-induced cystitis rabbit model [39]. PRP also has neuroprotective function by way of increasing the regeneration of axonal diameter and amount of Schwann cells [40]. In traumatic patients and traumatic animal models, injection of PRP into burn scar areas lessened inflammatory pain, and increased the activation of p-p38/NFκB signaling pathways which induced the activation of microglia and astrocytes [41,42].

The clinical utilization of PRP had been applied in several urologic diseases, including stress urinary incontinence (SUI) [43], recurrent bacterial cystitis [44], erectile dysfunction (ED), and IC/BPS [45]. Local injection of autologous PRP at anterior vaginal wall where mid-urethra locates is effective in treating women with SUI and the effect can last as long as 6 months post-treatment without significant adverse effect [43]. The effect of intravesical instillation with PRP was found to increase mitotic activity and significantly decrease bladder bleeding on recurrent bacterial cystitis in women [44]. PRP has also been shown to have a bacteriostatic activity [46]. The animal studies demonstrated that the administration of PRP could reduce infections with gram-negative bacterial (e.g., Escherichia coli) or Staphylococcus aureus [47,48]. The antimicrobial properties of platelet-rich gel can improve treatment of delayed healing of infections. Besides, PRP injection enhanced urothelial cell proliferation, cytoskeleton, as well as barrier protein expression in recurrent UTI [49]. Recently, a pilot study that perform repeated intravesical injections of autologous PRP could ameliorate bladder pain and increase bladder capacity in IC/BPS patients [39,45,50,51]. Clinical trial has demonstrated meaningful increased urinary IL-2 and IL-8 biomarker expressions, increased functional bladder capacity, and improvement in IC/BPS symptoms at 3-month follow-up in IC patients who received repeated monthly PRP intravenous injections for 4 months [52,53]. PRP injection could provide an optional therapy for IC/BPS patients who were refractory to conventional therapies. In a rabbit model of cyclophosphamide-induced cystitis, PRP was found to significantly stimulate angiogenesis and increase proliferation to improve bladder mucosal repair [44]. In a rat model of bilateral cavernous nerve injury, injection of PRP into the corpus cavernosum can promote nerve regeneration and erectile function recovery. The effect of PRP was also found to prevent the apoptosis of corporal smooth muscle cell.

The aim of this study was to investigate the pathophysiological mechanism of bladder dysfunction in KIC rat model and the underlying therapeutic effects of PRP on tissue remodeling. Accordingly, the present study was designed to explore the biological effect of PRP on bladder remodeling and tissue repair, including anti-inflammation, cell proliferation, angiogenesis, neurogenesis, myofibroblastic differentiation and production of extracellular matrix (ECM) to reverse the bladder damage in rats with KIC. We hope to develop a non-drug therapy directly generated from autologous blood without the risk of drug allergy.

## 2. Results

### 2.1. General Characteristics

Physical indicators, serum, urine parameters were shown in Table 1. There were no meaningfully difference in the amount of body weight, water intake, urine output, and serum level of ketamine and norketamine among different groups. Clinically, those ketamine abusers usually have body weight loss. In the present study, the body weight of the ketamine group was found to decrease slightly compared with the control group, but not statistically significant. Additionally, the ratio of bladder weight/body weight in the ketamine group was significantly increased compared to the control group. Moreover, urine samples from the ketamine, ketamine + PRP and ketamine + PPP groups showed increased levels of ketamine and noxzapine compared to the control group.

### 2.2. Effect of PRP Treatment Ameliorated Bladder Overactivity

**Figure 1:**
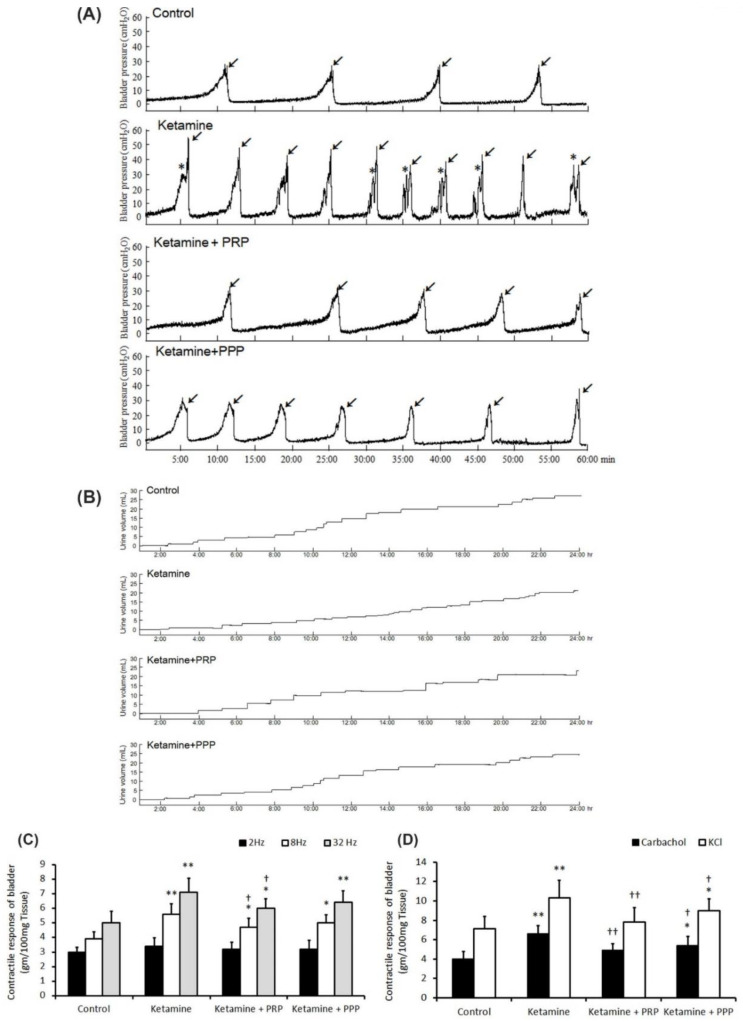
Cystometric parameter, voiding behavior and contractile responses. (**A**) Cystometric recordings performed for micturition pressure, voiding volume, and urinary frequency, including voiding contraction (arrows) and non-voiding contraction (asterisks). (**B**) Tracing analysis of voiding behavior for 24 h by metabolic cage. The ketamine group exhibited significantly increases in bladder micturition pressure, voiding contractions, non-voiding contractions and micturition frequency, whereas PRP and PPP treatment meaningfully ameliorated bladder micturition pattern and capacity. (**C**,**D**) Contractile responses of bladder strips and statistics data were examined by electrical field stimulation (2 Hz, 8 Hz and 32 Hz) (**C**), carbachol (**D**) and KCl (**D**). Data were expressed as means ± SD for *n* = 4, * *p* < 0.05; ** *p* < 0.01 versus the control group; ^†^ *p* < 0.05; ^††^ *p* < 0.01 versus the ketamine group.

The bladder function and voiding behavior were shown in Table 1 and Figure 1. The control group behaved steady and regular micturition patterns. The ketamine-treated rats behaved bladder overactivity with increased micturition frequency (arrows), increased non-voiding contraction (asterisks) and enhanced peak micturition pressure when comparing with the control group. On the contrary, both the ketamine + PRP group and the ketamine + PPP group showed meaningfully decreased urinary frequency, decreased peak micturition pressure, but increased bladder capacity and voiding interval when comparing to the ketamine group (Figure 1A and Table 1).

Additionally, voiding behavior with metabolic cage study revealed that ketamine treatment decreased bladder volume, but increased micturition frequency as compared with the control group (Figure 1B). On the contrary, both the ketamine + PRP group and the ketamine + PPP group had increased voiding volume and micturition interval as compared with the ketamine group. Taken together, the above findings revealed that ketamine injection increased voiding frequency and deteriorated bladder capacity, these phenomena are consistent with human ketamine cystitis patients. In contrast, PRP and PPP treatment significantly improved bladder capacity and ameliorated KIC related bladder overactivity.

### 2.3. Contractile Responses of Bladder Strips

Figure 1 revealed the contractile responses of bladder strips in response to electrical field stimulation (EFS) (Figure 1C), carbachol (Figure 1D), and KCl (Figure 1D) stimulation. The ketamine group showed stronger detrusor contractile responses to EFS at 8 and 32 Hz than the control group or the ketamine + PRP group (Figure 1C). Similar results were found in response to carbachol (Figure 1D) and KCl (Figure 1D). These findings proved that ketamine treatment enhanced detrusor muscle contractile activities in response to these stimulations, whereas PRP treatment reversed such changes.

### 2.4. PRP Regulated the Ketamine-Induced Pathological Modification

**Figure 2:**
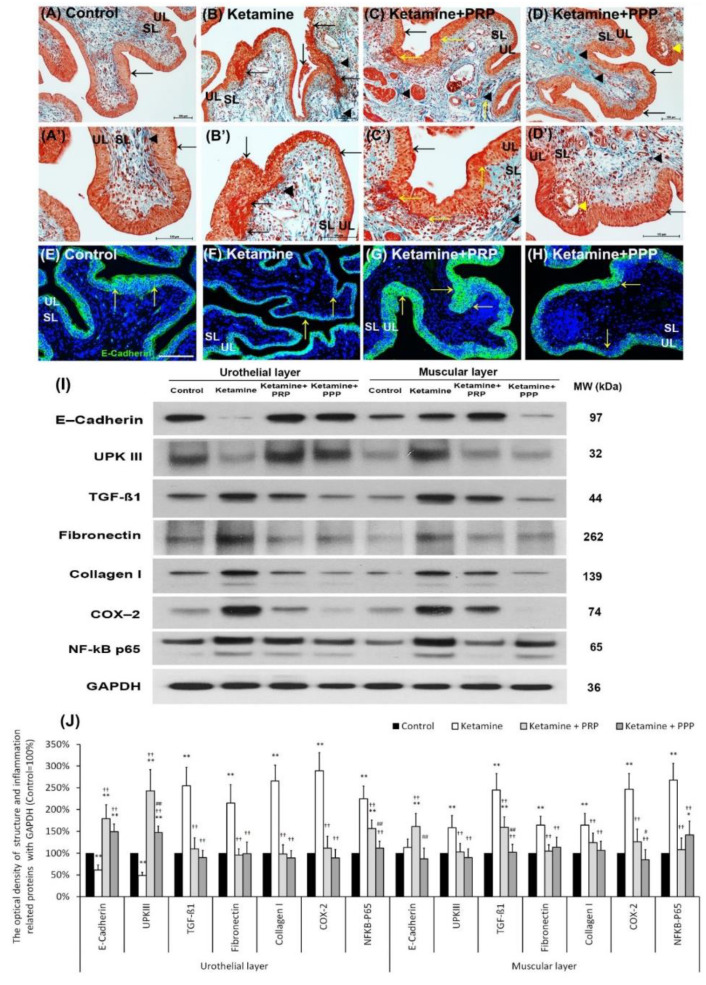
Therapeutic effect of PRP improved ketamine-induced pathological alteration by Masson’s trichrome staining, immunofluorescence and western blotting analysis. (**A**–**D**’) Bladder pathological features of the control group (**A**,**A’**), the ketamine group (**B**,**B’**), the ketamine + PRP group (**C**,**C’**) and the ketamine + PPP group (**D**,**D’**). Masson’s trichrome stain showed red-stained smooth muscle, navy blue-stained nucleus, green-stained collagen. In the control group (**A**,**A’**), there were three to five layers of the urothelial layer (UL) and only scattered collagen (yellow arrow) distributed in the suburothelial layer (SL; lamina propria). In the ketamine group (**B**,**B’**), the morphology was characterized by thinner layer of urothelial cells, hemorrhage (Black arrows), much collagen accumulation (Black arrowheads) and increased bladder fibrosis (yellow arrows). In contrast, the pathological features of the ketamine + PRP group (**C**,**C’**) and the ketamine + PPP group (**D**,**D’**) showed recovered ketamine-induced bladder damages by increasing thicker layer of urothelium (Black arrows) and reducing interstitial fibrosis (Black arrowheads) when comparing to the ketamine group. Especially, there was a vacuolation beneath urothelial layer (Yellow arrowhead) in the ketamine + PPP group. (**E**–**H**) The expression of cell-adhesion protein E-cadherin by immunostaining was shown. In the control group (**E**), the E-cadherin staining was found in urothelial intercellular junctions. In contrast, there was less E-cadherin staining distribution in thin urothelium of the ketamine group (**F**), but the immunostaining of the ketamine+ PRP group (**G**) and the ketamine+ PPP group (**H**) were enhanced in urothelium. (**I**,**J**) Western blotting analysis was performed to evaluate the protein levels of urothelial structure (E-cadherin and UPKIII) and bladder inflammation (TGF-ß1, COX-2 and NF-κB), interstitial fibrosis (fibronectin and type I collagen). Both the inflammatory and fibrosis markers (TGF-ß1, fibronectin, type I collagen, COX-2 and NF-κB) were meaningfully elevated in UL and ML of the ketamine group when comparing to the control group. Moreover, the proteins were markedly decreased in the ketamine + PRP group and the ketamine + PPP group when comparing to the ketamine group. Results were normalized as the control = 100%. UPKIII, uroplakin III; TGF-ß1, transforming growth factor ß1. Data were expressed as means ± SD for *n* = 6, * *p* < 0.05; ** *p* < 0.01 versus the control group; ^††^ *p* < 0.01 versus the ketamine group; ^#^ *p* < 0.05; ^##^ *p* < 0.01 versus the ketamine + PRP group.

The therapeutic effect of PRP instillation on the ketamine-induced pathological alteration was shown in Figure 2A–D’. In comparison to the control group (Figure 2A,A’), the morphology of the ketamine group expressed thinner urothelial layer (UL), loss of superficial cells (epithelial loss), erythematous mucosa (hemorrhage), mucosa ulceration (black arrows) and more collagen accumulation (black arrowheads) of suburothelial layer (SL) (Figure 2B,B’). Furthermore, morphological assessment of the ketamine + PRP group (Figure 2C,C’) and the ketamine + PPP group (Figure 2D,D’) compared to the ketamine groupshowed recovery of ketamine-associated bladder injury by increasing thicker layer of UL and regulating interstitial fibrosis (Figure 2B,B’). In particular, there are many red blood cells clustered in UL and SL (yellow arrows) in the ketamine + PRP group (Figure 2C,C’). These results revealed that the effect of PRP administration may improve mucosal integrity, reduce hemorrhage and modulate interstitial fibrosis to improve bladder repair in KIC model.

The distribution of E-cadherin (a cell-adhesion marker) in the bladder tissue was also examined (Figure 2E–H). In Figure 2E, the E-cadherin staining in control group appeared only in urothelial intercellular junctions but not in the suburothelium. In contrast, the distribution of E-cadherin staining in the urothelium was less in the ketamine group (Figure 2F) compared to the control group, but the immunostaining of E-cadherin in the ketamine + PRP group (Figure 2G) and the ketamine + PRP group (Figure 2H) was enhanced when comparing to the ketamine group. More importantly, the E-cadherin immunostaining in the ketamine + PRP group was recovered to the control group level.

The protein levels of urothelial cell-adhesion marker (E-cadherin) and differentiated marker (UPKIII) as well as inflammation and fibrosis markers (TGF-ß1, fibronectin, type I collagen, COX-2 and NF-κB P65) were evaluated by Western blotting analysis (Figure 2I,J). According to the data, the urothelial expressions of E-cadherin and UPKIII were meaningfully decreased in the ketamine group as compared with the control group. However, the markers in UL and ML of the ketamine + PRP group as well as UL of the ketamine + PPP group were increased significantly compared to the ketamine group (Figure 2I,J). Besides, the inflammatory and fibrosis markers were markedly increased in the ketamine group when comparing with the control group. On the contrary, the markers were noticeably decreased in UL and ML of the ketamine + PRP group and the ketamine + PPP group compared to the ketamine group. These observations implied that weekly PRP and PPP treatment can cause inflammatory reaction and modulate interstitial fibrotic progression. Based on the results of morphological evaluation and western blotting analysis, the ketamine group meaningfully aggravated bladder pathological injury and interstitial fibrosis. Conversely, PRP treatment modulated the fibrotic biosynthesis, improved urothelial barrier and ameliorated the bladder injury.

### 2.5. PRP Improved Urothelial Proliferation and Tight Junction Function

**Figure 3:**
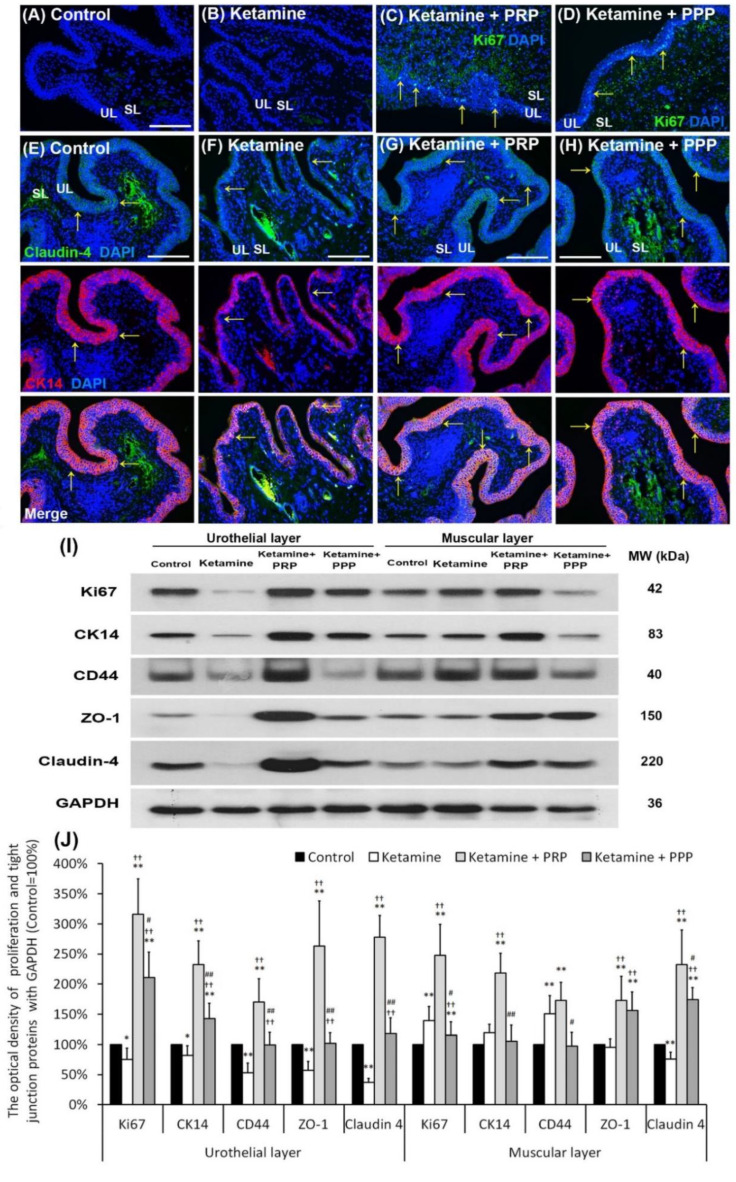
PRP strengthened urothelial proliferation and junction-associated protein expression. The expressions of proliferating and differential marker (Ki67, CK14 and CD44) markers and urothelial tight junction (Claudin-4 and ZO-1) were assessed by immunofluorescence evaluation (**A**–**H**) and western blotting analysis (**I**,**J**). (**A**–**D**) The staining of Ki67 (a proliferation marker) was less expression in the bladder tissues of the control group (**A**), and the ketamine group (**B**). On the contrary, the Ki67 immunostaining was expressed in the urothelial basal layer in the ketamine + PRP group (**C**) and the ketamine + PRP group (**D**). (**E**–**H**) The co-staining of claudin-4 and CK14 was widely expressed in the urothelial layer. Double-labeled analysis of Claudin-4 (fluorescein isothiocyanate; green, upper panels) and CK14 (rhodamine; red, lower panels) was widely expressed in the urothelial layer in the control group (**E**). The co-staining was restricted to the thin and disrupted urothelium in ketamine group (**F**). Conversely, the staining of the ketamine + PRP group (**G**) and the ketamine + PPP group (**H**) was markedly distributed in the urothelial basal layer compared to the ketamine group (**F**). Nuclear DNA was labeled with DAPI (blue). (**I**,**J**) Western blotting analysis was used to quantified the percentage of Ki67, CK14, CD44, Claudin-4 and ZO-1. The protein levels were meaningfully enhanced in the ketamine + PRP group when comparing to the ketamine group. Results were normalized as the control = 100%. CK, cytokeratin. Data were expressed as means ± SD for *n* = 6, * *p* < 0.05; ** *p* < 0.01 versus the control group; ^††^ *p* < 0.01 versus the ketamine group; ^#^ *p* < 0.05; ^##^ *p* < 0.01 versus the ketamine + PRP group.

Whether impaired mucosal integrity altered voiding patterns and urodynamic responses were shown in Figure 3. The Ki67 immunostaining was less distribution in the bladder tissues of the control group and the ketamine group. In contrast, the Ki67 immunostaining was obviously distributed in the urothelial basal layer in the ketamine + PRP group and the ketamine + PPP group (Figure 3A–D). Besides, in the control group, the co-staining of CK14 and Claudin-4 was widely expressed in the urothelial basal layer. In the ketamine group, the co-staining was restricted to the thin, disrupted urothelium and tight junction. However, the co-staining of the ketamine + PRP group and the ketamine + PPP group was noticeably expressed in the urothelial basal layer when comparing to the ketamine group (Figure 3E–H). Particularly, the immunostaining of the ketamine + PRP group was stronger than the ketamine + PPP group. The increased proliferation index was assessed by CK14-positive (CK14^+^) and Ki67-positive (Ki67^+^) immunostaining in urothelial basal layer (data not shown). These findings suggested that mitotic effect of PRP stimulated mucosal proliferation in the urothelial basal layer to improve mucosal regeneration.

The protein levels of proliferation markers (Ki67, CK14 and CD44) and urothelial tight junction markers (Claudin-4 and ZO-1) (Figure 3I,J) were investigated by Wester blotting analysis. Both the proliferation and urothelial tight junction association protein levels were noticeably decreased in the UL of the ketamine group when comparing to the control group. However, those protein levels were meaningfully increased in the UL and muscular layer (ML) of the ketamine + PRP group compared to the ketamine group and the ketamine + PPP group. Moreover, in comparison to the UL of the ketamine + PRP group, the expression of CD44 (HA receptor) was meaningfully decreased in the UL of the ketamine group and the UL and ML of the ketamine + PPP group (Figure 3I,J). These findings demonstrated that PRP treatment could increase bladder regeneration via cell proliferation, differentiation and increasing HA expression.

### 2.6. PRP Improved Bladder Angiogenesis

**Figure 4:**
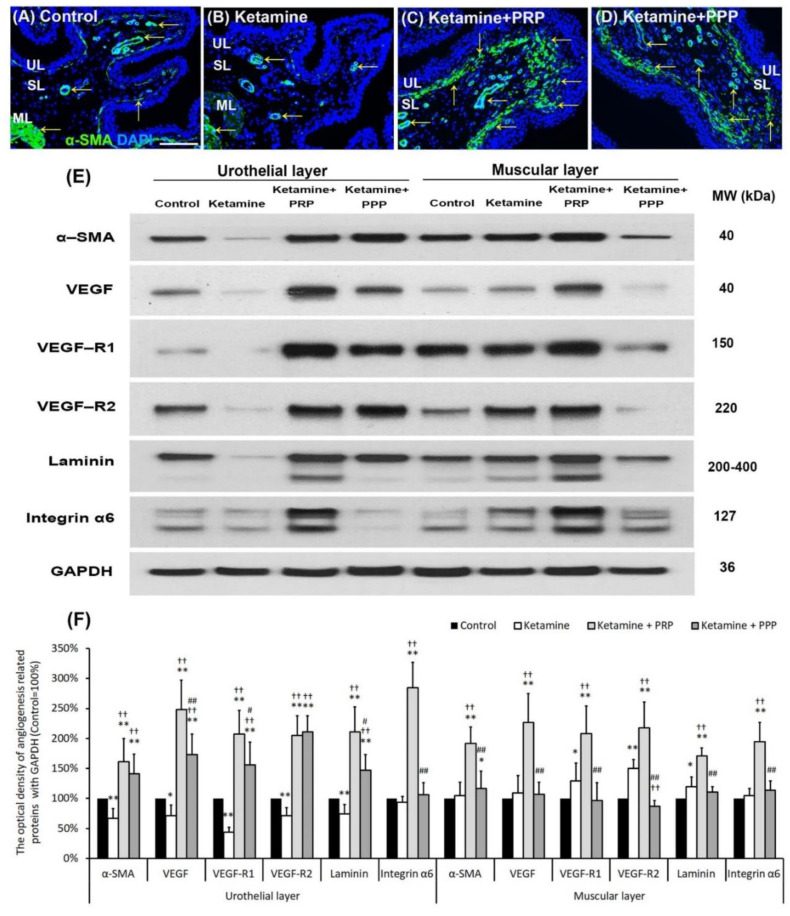
Bladder angiogenic remodeling was triggered by PRP treatment. The angiogenesis markers were analyzed by immunostaining (**A**–**D**) and western blotting analysis (**E**,**F**). (**A**–**D**) The α-SMA immunostaining was widely expressed in smooth muscle of microvessels beneath urothelial basal layer and vessels in suburothelial layer (SL) and muscular layer (ML) in the control group (**A**). In the ketamine group (**B**), the staining of α-SMA was reduced in the SL compared to the control group. However, the staining level in the ketamine + PRP group (**C**) and the ketamine + PPP group (**D**) were increased in SL and beneath urothelial basal layer compared to the ketamine group. Particularly, the level of the ketamine + PRP group in SL and ML was much stronger than the ketamine + PPP group. (**E**,**F**) Western blotting analysis was used to quantified the percentage of angiogenesis related proteins, including α-SMA, VEGF, VEGF-R1, VEGF-R2 (VEGF receptors), Laminin and Integrin-α6 (Laminin receptor). The protein concentration in the ketamine group was much lower than the control group. Conversely, the expressions of angiogenesis markers were significantly increased in the ketamine + PRP group when comparing to the ketamine group and the ketamine + PPP group. Therefore, PRP altered bladder angiogenic remodeling. VEGF, vascular endothelial growth factor. Results were normalized as the control = 100%. Data were expressed as means ± SD for *n* = 6, * *p* < 0.05; ** *p* < 0.01 versus the control group; ^††^ *p* < 0.01 versus the ketamine group; ^#^ *p* < 0.05; ^##^ *p* < 0.01 versus the ketamine + PRP group.

Whether PRP therapy altered bladder angiogenesis in the pathogenesis of KIC were shown in Figure 4. In the control group (Figure 4A), immunostaining showed that the α-SMA was broadly expressed in microvessels beneath urothelial basal layer, suburethral layer (lamina propria) and ML. In the ketamine group, the α-SMA expression was reduced in microvessels almost in whole layer of bladder when comparing to the control group. However, the expression in the ketamine + PRP group and the ketamine + PPP group was enhanced in SL when comparing to the ketamine group (Figure 4B–D).

To elucidate the angiogenic effect of PRP instillation, the levels of angiogenesis associated proteins were quantified by western blotting analysis (Figure 4E,F). The expressions of these angiogenic proteins in the UL of the ketamine group were significantly reduced compared to the control group, with the exception of integrin-α6. Moreover, the protein expressions were obviously increased in the UL of the ketamine + PRP group when comparing to the ketamine group. The expression levels of VEGF, VEGF-R1, laminin and integrin-α6 in the UL of the ketamine + PRP group were stronger than the other group. Moreover, in the ML of the ketamine + PRP group, the expressions of these angiogenesis associated proteins were also significantly increased compared to the other group. Similar expression occurred in ML between the control group and the ketamine + PPP group (Figure 4E,F). According to the above data, PRP treatment promoted bladder circulation by increasing capillary density and VEGF expression. PRP could promote angiogenic potential for bladder repair through laminin/integrin-α6 and VEGF/VEGF-R signaling pathways in KIC. More importantly, PRP might prevent fibroblast-myofibroblast transition and inflammatory fibrosis via VEGF/VEGF-R-mediated inhibition.

### 2.7. PRP Treatment Increased Bladder Nerve Regeneration

**Figure 5:**
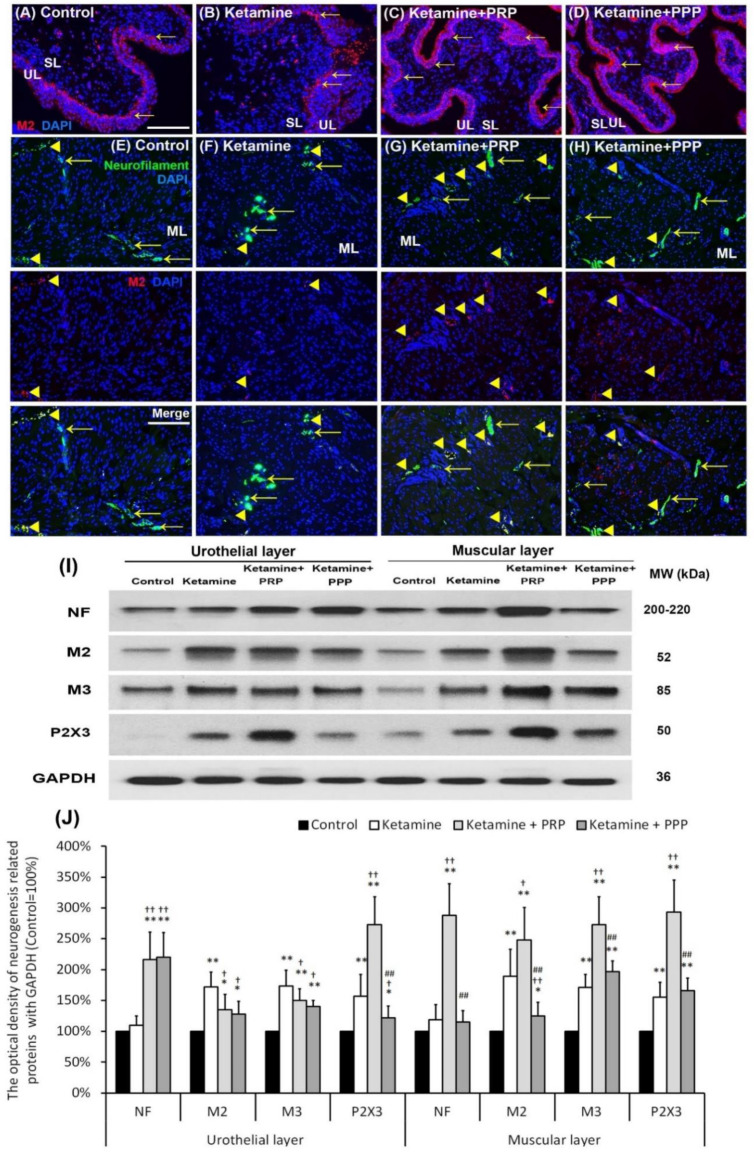
PRP treatment increased bladder nerve regeneration. The neurogenesis markers were analyzed by immunostaining (**A**–**H**) and western blotting analysis (**I**,**J**). (**A**–**D**) The M2 immunostaining was widely stained in the urothelial basal layer in the control group (**A**). In the ketamine group (**B**), the M2 immunostaining (red; arrows) was shown in the basal layer and disrupted urothelium in the ketamine group. Besides, the M2 immunostaining was markedly distributed in the urothelial layer and intermediate layer in the ketamine + PRP group (**C**) and the ketamine + PPP group (**D**). (**E**–**H**) The co-labeling of M2 and NF (yellow arrowheads) in the ketamine + PRP group was obviously expressed in the ML when comparing to the ketamine group and the ketamine + PPP group. Nuclear DNA was labeled with DAPI (blue). (**I**,**J**) Western blotting analysis for the bladder neurofilament (NF), muscarinic (M2 and M3) and purinergic (P2X3) receptors were performed in each group. Quantifications of the percentage of NF, M2, M3 and P2X3 expressions to β-actin were shown. The expressions were significantly increased in the ketamine group when comparing to the control group. However, administration with PRP meaningfully increased the expression levels of these receptors in UL and ML of bladder. Results were normalized as the control = 100%. Data were expressed as means ± SD for *n* = 6, * *p* < 0.05; ** *p* < 0.01 versus the control group; ^†^ *p* < 0.05; ^††^ *p* < 0.01 versus the ketamine group; ^##^ *p* < 0.01 versus the ketamine + PRP group.

The therapeutic effect of PRP instillation on bladder intramural nerve damage in KIC was shown in Figure 5. The muscarinic and purinergic receptors increased bladder afferent nerve activity and detrusor activity. In the control group, the M2 expression (arrows) mainly spread in the basal layers of the urothelium. Besides, the M2 immunostaining (arrows) was shown in the basal layer and disrupted urothelium of bladder tissues in the ketamine group. In contrast, the M2 immunostaining was markedly distributed in the urothelial layer and intermediate layer in the ketamine + PRP group and the ketamine + PPP group (Figure 5A–D).

Whether altered voiding pattern and detrusor activity were associated with bladder afferent nerve activity were shown in Figure 5E–H. In the control group, the M2 (red) and NF (green) co-staining (Arrowheads) was distributed in the ML (Figure 5E). The co-labeling of M2 and NF in the ketamine + PRP group was significantly expressed in the ML when comparing to the ketamine group and the ketamine + PPP group (Figure 5F–H). Western blotting analysis and statistical comparisons were performed to determine the protein levels of NF, M2, M3 and P2X3 (Figure 5I,J). The protein levels in the UL and ML of the ketamine group were noticeably increased when comparing to the control group. Especially, the levels of NF, M2, M3 and P2X3 meaningfully were enhanced in the ML of the ketamine + PRP group when comparing to the ketamine group. These results confirmed that ketamine induced hyperactivity bladder via intramural nerve damage and muscarinic receptor as well as purinergic receptor overexpression. On the contrary, PRP treatment increased bladder nerve regeneration and receptor expression.

### 2.8. PRP Reduced Bladder Oxidation and Enhanced Anti-Inflammation Enzymes

**Figure 6:**
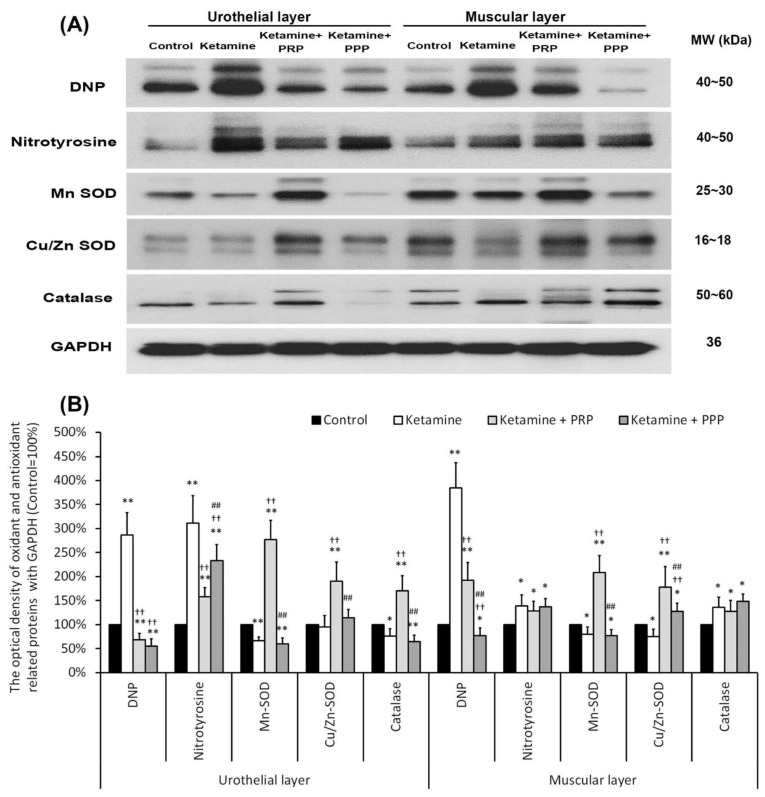
PRP treatment reduced bladder oxidation and enhanced anti-inflammation enzymes expression. Western blotting analysis for oxidative markers (DNP and nitrotyrosine) and antioxidant enzymes (MnSOD, Cu/ZnSOD and catalase) expressions were measured in each group (**A**). Quantifications of the percentage of oxidative markers and antioxidant enzymes expressions to β-actin were shown (**B**). The expressions of DNP and nitrotyrosine were significantly increased in the ketamine, but were decreased in the ketamine + PRP group and the ketamine + PPP group. The expressions of antioxidant enzymes were meaningfully increased in the ketamine + PRP group. Conversely, administration with PRP meaningfully decreased the expression levels. Results were normalized as the control = 100%. Data were expressed as means ± SD for *n* = 6, * *p* < 0.05; ** *p* < 0.01 versus the control group; ^††^ *p* < 0.01 versus the ketamine group; ^##^ *p* < 0.01 versus the ketamine + PRP group.

The expressions of oxidative stress markers (DNP and nitrotyrosine) and antioxidant enzymes (MnSOD, Cu/ZnSOD and catalase) were shown by western blotting analysis in Figure 6. The expression levels of DNP and nitrotyrosine were increased in the UL and ML of the ketamine group when comparing to the control group. The UL and ML levels of DNP and nitrotyrosine were meaningfully reduced in the ketamine + PRP group and the ketamine + PPP group compared to the ketamine group. Besides, the expressions of antioxidant enzymes were meaningfully reduced in the UL and ML of ketamine group compared to the control group. Conversely, the expression levels of antioxidant enzymes in the ketamine + PRP group was stronger than the other group. The above observations revealed that PRP treatment regulated the oxidative stress and antioxidant enzymes in the KIC bladder. These findings demonstrated that ketamine exacerbated bladder oxidative damage, whereas PRP offered a beneficial effect on lessening ketamine-induced oxidative damages.

### 2.9. A Proposed Diagram for the Effect of PRP on Enhancing Anti-Inflammation, Promoting Cell Proliferation, Altering Angiogenesis and Neurogenesis to Improve KIC-Induced Bladder Overactivity

**Figure 7:**
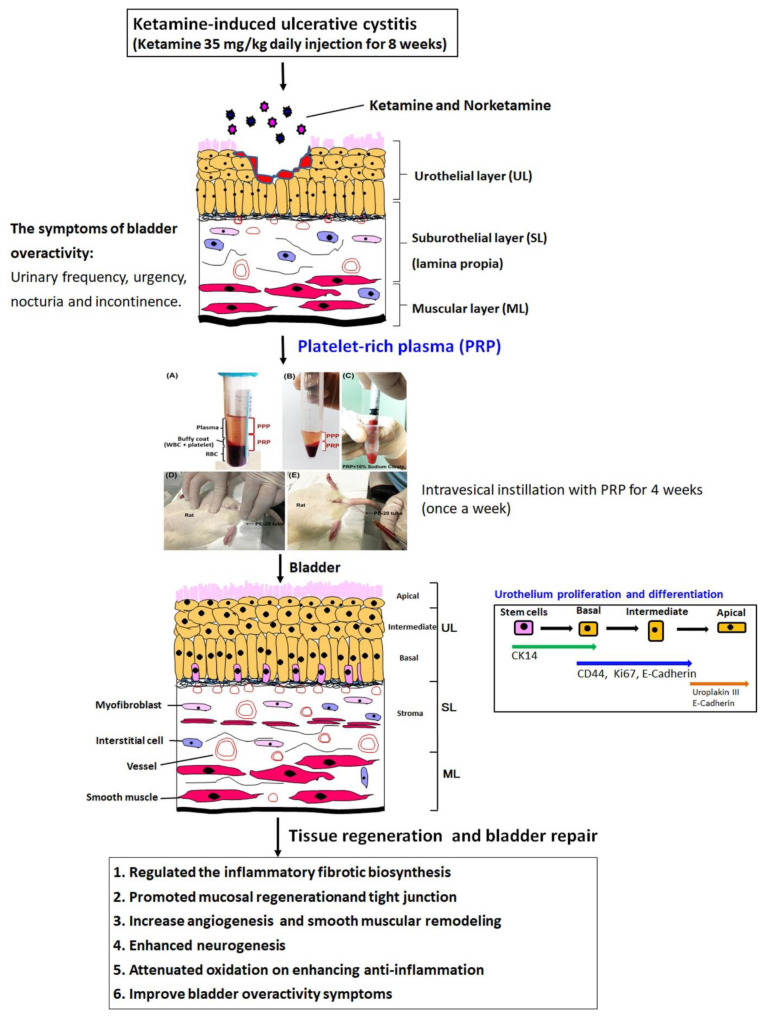
A proposed diagram for the potential effect of PRP on improving the KIC symptoms. The ketamine group meaningfully exacerbated bladder pathological damage and interstitial fibrosis through NF-κB/COX-2 signaling pathways. In contrast, intravesical instillation with PRP for 4 weeks regulated the inflammatory fibrotic biosynthesis, modulated fibroblast-myofibroblast transition, enhanced anti-inflammation, promoted cell proliferation and altered angiogenesis and neurogenesis to improve bladder function and ameliorate the OAB symptoms.

A brief diagram was proposed for the therapeutic effect of PRP for bladder overactivity induced by KIC in rat model (Figure 7). This proposed model established a long-term ketamine abuse and identified possible mechanisms of detrusor overactivity. Rats treated with 35 mg/kg ketamine daily for 8 weeks showed increased voiding frequency and decreased bladder capacity. These symptoms were observed in human ketamine abusers. Accordingly, the ketamine group exacerbated bladder pathological damage and interstitial fibrosis through NF-κB/COX-2 signaling pathways. In contrast, intravesical instillation with PRP for 4 weeks regulated the inflammatory fibrotic biosynthesis, modulated fibroblast-myofibroblast transition, enhanced anti-inflammation, promoted cell proliferation and increased angiogenesis and neurogenesis to improve bladder function. PRP could promote angiogenic potential for bladder repair through laminin/integrin-α6 and VEGF/VEGF-R signaling pathways in the pathogenesis of KIC. Besides, the neuroprotective effect of PRP stimulated neurofilament, muscarinic and purinergic receptors to ameliorate ketamine-induced bladder dysfunction.

## 3. Discussion

The present investigation revealed that ketamine injection lessened bladder capacity and voiding function, whereas PRP treatment meaningfully improved bladder capacity and ameliorated OAB symptoms induced by ketamine. Based on the data of morphological evaluation and western blotting analysis, these observations demonstrated that the ketamine group meaningfully exacerbated bladder pathological injury and interstitial fibrosis through NF-κB/COX-2 signaling pathways. In contrast, PRP enhanced the effect of TGF-ß released by platelets and regulated the inflammatory fibrotic biosynthesis, modulated fibroblast-myofibroblast transition to ameliorate bladder injury in rats with KIC. Moreover, the mitotic effect of PRP improved mucosal regeneration by stimulating CK14-positive (CK14^+^) and Ki67-positive (Ki67^+^) cells in the urothelial basal layer assessed and increasing the expressions of CK14, Ki67 and CD44. Besides, the expressions of α-SMA, VEGF, VEGF receptor (VEGF-R1 and VEGF-R2), laminin, and laminin receptor (integrin-α6) in the ketamine group were significantly suppressed compared to the control group, but PRP could promote angiogenic potential in bladder UL and ML through laminin/integrin-α6 and VEGF/VEGF-R signaling pathways. Moreover, the toxicity of ketamine and noreketamine induced nerve fiber degeneration and caused hyperactivity bladder via intramural nerve damage and muscarinic receptor overexpression. However, the neuroprotective effect of PRP stimulated neurofilament regeneration and activated muscarinic and purinergic receptors to restore the ketamine-induced bladder dysfunction. These findings suggested that the effect of PRP regulated the inflammatory fibrotic biosynthesis, promoted mucosal regeneration, attenuated oxidation, enhanced angiogenesis and neurogenesis to improve bladder function and ameliorate the OAB symptoms in rats with KIC.

The present studies proposed that PRP contains not only a high level of platelets, but also many growth factors such as TGF-ß, PDGF, VEGF, EGF, and IGF [14,15]. The effects of TGF-ß1 promoted suburothelial and interstitial fibroblasts differentiated into myofibroblasts, characterized by the α-SMA expression to increase production of type-I collagen and ECM remodeling [54,55]. Myofibroblasts produce abundant ECM proteins and play an important role in the remodeling of damaged connective tissues during tissue repair [54]. Based on the present data, intravesical instillation with PRP not only may cause a mild inflammatory response, but also promote mucosal regeneration and enhance angiogenesis and neurogenesis to improve bladder function and ameliorate the OAB symptoms. In spite that PRP induces minor inflammation in bladder, it also has advantages.

The present studies hope to develop a non-drug autologous PRP therapy for KIC, which is generated from autologous blood and causes no rejection, and is superior to synthetic materials. However, the main limitation of this animal study was the difficulty in obtaining a sufficient volume of uncoagulated blood from the rats’ tail vein. While only 0.2 mL of PRP could be gathered because only 1 mL of blood can be obtained from rats. In human, 20 mL of blood drawn from the anterior tibial vein can very easily provide 6–8 mL of PRP. For example, the total bladder capacity of the rat was approximately 1.8–2.5 mL, and 0.3–0.4 mL was used for intravesical instillation. The animals’ bladders were emptied prior to intravesical instillation. 0.3 mL mixed with 0.1 mL of 10% calcium chloride solution and 0.2 mL of PRP or PPP were injected into bladder. Following, the catheters were removed after 30 min to ensure that they did not invalidate subsequent PRP perfusion. Once PRP is triggered by the addition of autologous thrombin and CaCl_2_ for activation, growth factors secretion begins within 10 min; approximately 95% of all growth factors are secreted within 1 h [56]. A typical blood specimen consists of 1% white blood cells, 6% platelets and 93% red blood cells. The PRP treatment is used to reverse the ratio of red blood cells to platelets by reducing red blood cells to 5% and more importantly concentrating platelets containing a powerful mixture of growth factors to 94%. Most studies have suggested that increasing platelets by at least five times the normal concentration (about 1 million platelets/μL) could enhanced bladder restorative efficacy [57,58], whereas higher concentrations did not show further improvement of wound healing. However, despite the small volume, a significant effect was observed in the bladder urothelium. PRP repeated instillations would require more thrombocytes and a larger volume of growth factors. The PRP preparation techniques and application protocols need to be standardized in order to perform the therapeutic effect. In addition, the variability in platelet concentration, PRP dosage, equipment and techniques used may alter the characteristics of platelet degranulation and thus affect clinical outcomes. The direct contact between PRP and the damaged urothelium, as well as the subsequent release of tissue factors and the paracrine effects of these factors, can be considered as initiating cell proliferation in the basal membrane. Previous study has shown that up to 70% of the mediator is sufficient to release within 10 min of contact between PRP and damaged mucosa [59]. The purpose of this study was to evaluate whether PRP could accelerate the regeneration of the bladder urothelium.

Current approaches to the treatment of KIC reflected the deficiency of a universally accepted mechanism. Anticholinergics, anti-inflammatory drugs, intravesical glycosaminoglycan preparations, pentosan polysulfate, and other supportive therapies have limited applications. Therefore, mechanism understanding of KIC is the prerequisite for development of effective therapies. Previous data showed that ketamine inhibited the L-type voltage-gated calcium channel Cav1.2, which caused functional and pathological changes in smooth muscle. The activation of this channel can reverse these pathological changes [7]. Our previous study showed that ketamine treatment enhanced bladder interstitial fibrosis, alternated in micturition patterns, accelerated macrophage infiltration and initiated up-regulation of COX-2, and induced expression of endothelial nitric oxide synthase and nitric oxide synthase, whereas COX-2 inhibitor reduced the intensity of fibrosis in a rat model [8,60]. Moreover, promoter-deletion analysis revealed that the expression of COX-2 via transcription factor NF-κB p65 was involved in the inflammatory signaling in KIC of the rat bladder. The methylation of CpG sites in the COX-2 promoter through NF-κB transcriptional regulation affected the COX-2 transcriptional expression in a KIC animal model [61]. Activation of NF-κB with KIC induced various proinflammatory cascade, as cytokines (TNF-α, IL-1, IL-6, IL-8, and IL-12), chemokines, and thus plays important roles in the immune and inflammation processes. Our data also revealed that treatment with ketamine significantly increased phosphorylation of Akt, induced mitochondrial and organelle swelling and degeneration, inhibited angiogenesis, damaged endothelial cells, induced mast cell and eosinophil-mediated inflammation, enhanced interstitial fibrosis, and led to bladder overactivity [62]. The present study suggested that the effect of PRP contained high level of platelets and numerous growth factors regulated the inflammatory fibrotic biosynthesis, promoted mucosal regeneration, attenuated oxidation, enhanced angiogenesis and neurogenesis to improve bladder function and ameliorate the OAB symptoms in rats with KIC.

Parasympathetic nerves stimulation of both M2 and M3 muscarinic receptors by acetylcholine as well as of purinergic receptor (P2X) by ATP triggers contraction of bladder detrusor muscle. Animal studies suggested that neurodegeneration is a potential long-term risk of ketamine anesthetics in neonatal and young pediatric patients. In rats with normal spinal cords, PRP induced activation of microglia and astrocyte and expression of ICAM-1 and PDGF-ß. More importantly, the delivery of PRP has neurotrophic effects on axonal growth and regeneration after spinal cord injury [63]. For example, at 8 weeks mice treated with ketamine (100 mg/kg) showed increased voiding frequency, reduced inter-contraction interval, and decreased bladder capacity and poor bladder compliance on urodynamics. These symptoms also develop in human ketamine abusers. Furthermore, immunohistochemical staining revealed increased P2X1 receptor distribution in ketamine treated bladders while M2 and M3 receptor distribution was unaffected. Increased non-cholinergic contractions and P2X1 receptor expression in the KIC bladder indicated that dysregulation of purinergic neurotransmission may trigger detrusor overactivity in cases of ketamine induced bladder dysfunction [6]. Our present data suggested immunostaining and western blotting analysis revealed increases in the expression of M2, M3 and P2X3 receptors in ketamine treated bladders when comparing to the control group, except axon neurofilament. However, the strong staining of neurofilament, M2, M3 and P2X3 receptors in the ketamine + PRP group was shown in UL and ML, when comparing to the control group and the ketamine group in Figure 5. In animal models, the PRP injection promoted the axon regeneration and enhanced the peripheral nerve regeneration in rats after spinal cord contusion and facial nerve transaction [64,65]. Nevertheless, Cho et al. applied PRP and mesenchymal stem cells (MSCs) to facial nerve axotomy, suggesting that PRP and MSCs promoted peripheral nerve regeneration in acute nerve injury [66]. Furthermore, by using a cerebrospinal co-culture system, Takeuchi et al. concluded that PRP enhanced the axonal growth in the bladder tissues by regulating the expression of EGF, IGF-1 and VEGF [67]. Our findings suggested therapeutic effect of PRP stimulated angiogenesis, enhanced neuronal regeneration and improved bladder overactivity in the pathogenesis of KIC in a rat model.

Angiogenesis is a complex process including endothelial cells, extracellular matrix (ECM) components and angiogenic factors. Angiogenesis after KIC is thought to be an endogenous protective response [68,69]. Therefore, microvasculature is a therapeutic target for neuroprotection after KIC. Besides, PRP is rich in angiogenic factors and may act as a modulator of the inflammatory response. Previous study demonstrated that PRP prevented myofibroblast generation and pathological angiogenesis via VEGF/VEGFR-1-mediated signaling to antagonize TGF-β1/Smad3 signaling to inhibit inflammatory fibrosis [70]. Moreover, the blockade of VEGF/VEGFR-1 mediated signaling blocked the inhibitory effect of PRP on TGF-β1-induced fibroblast-myofibroblast transition and downregulation of Smad3 expression [70]. Our data suggested that PRP increased the expressions of TGF-β1, fibronectin, type I collagen, α-SMA, VEGF, VEGF-R1, VEGF-R2, laminin and integrin-α6 to promote angiogenesis. The present study demonstrated that triggered angiogenesis by PRP administration after KIC was associated with augmented neurogenesis (Figure 3 and Figure 4). In addition, PRP could promote angiogenic potential for bladder repair through laminin/integrin-α6 and VEGF/VEGF-R signaling pathways in the pathogenesis of KIC. Besides, PRP might modulate fibroblast-myofibroblast transition and inflammatory fibrosis via VEGF/VEGFR mediated inhibition. However, the effect of other angiogenic factors in the PRP on KIC requires further investigation in future studies.

The antibacterial property and mechanism of PRP are not yet fully understood. PRP is a centrifuged autologous concentrate enriched platelet. The activator of PRP is a mixture of calcium chloride and thrombin, producing a platelet-rich gel. By concentrating platelets, higher levels of growth factors stimulated the healing processes. The effects of platelets have many functions in the antimicrobial host defense systems [71,72], including direct interaction with microorganisms, participate in antibody-dependent cell cytotoxicity against microbial pathogens, and contribute to the clearance of pathogens from the bloodstream [71,72,73]. Antimicrobial activities were dose-dependent. PRP also generated antimicrobial oxygen metabolites (e.g., hydroxyl free radicals, hydrogen peroxide, and superoxide) [71,72,73]. Yeaman et al. [73] suggested antimicrobial polypeptides from thrombin released materials and acid extracts of platelets. These findings indicated that the platelet concentration has a direct relationship with the antimicrobial effect. Combining our results with previous studies, PRP not only improves recurrent bacterial cystitis but also ketamine-induced ulcerative cystitis.

Effects of autologous PRP improved inflammatory infiltration and increased angiogenesis and collagen deposition using PRP-coated sutures in a rabbit model [74]. PRP also had significant effect in treating osteoarthritis [75,76] and bone healing [38]. The efficacy of different autologous platelet concentrates in animal models, including small ruminants, dogs and mini-pigs might positively affect bone regeneration. However, animal species, platelet and growth factors concentration, type of bone defect and the platelet concentrate used appear to influence their efficacy in bone healing [38]. Recently, PRP was also used in ophthalmology, odontal diseases and hair loss [36,77,78,79]. Alio et al. used PRP to treat dormant corneal ulcers with great outcome, 65.1% of patients improved their visual acuity [80]. Additionally, the effects of autologous platelet-rich fibrin in post-extraction alveolar sockets could be able to stimulate the natural process of tissue healing and regeneration of post-extraction sites in dogs with spontaneous periodontal disease [36]. In some studies, PRP was used for post-extraction sockets and bone regeneration after teeth extraction with significant improvement [81,82]. Additionally, more than three injections of the autologous non-activated platelet-rich plasma (A-PRP) and calcium-activated platelet-rich plasma (AA-PRP) into scalps increased the activation of Bcl-2 protein (anti-apoptotic regulator), CD31 (Platelet endothelial cell adhesion molecule-1), VEGF and Akt signaling pathways, leading to improving the dermal papilla cells survival and proliferation during the hair cycle, and ultimately to improved hair count and density in both male and female patients with androgenetic alopecia [77,78,79,83,84,85,86,87].

Clinically, ketamine abuser may cause bladder overactivity, inducing increased urinary urgency, frequency, nocturia, dysuria, suprapubic pain and occasionally gross hematuria [1,2,3]. Similar findings were shown in human patients and animal models. In our studies, a dose of 35 mg·kg^−1^·day^−1^ for 8 weeks was previously shown to induce rat ketamine cystitis with voiding dysfunction. Urine examination and histological study of bladder by Masson’s trichrome stain found that about 60% of rat had bladder hematuria and bladder hemorrhage. Such bladder hemorrhage and hematuria might be correlated with KIC severity, such as number of non-voiding contractions (without urine leakage during bladder infusion). Besides, these differences may be due to different dose, duration and individual physical characteristics.

There are some limitations in present study. This is a preclinical trial in which safety and efficacy are primarily assessed. However, the predictive validity, which allows the response in animals to be compared with the human response, is generally overestimated. Besides, animal species, platelet and growth factors concentration, PRP injection site and residence time, type of platelet concentrate as well as of tissue defect, and standardized procedure including aseptic technique, centrifuge speed and the quality of centrifuge tube used seemed to influence the efficacy. Further clinical study is necessary to find out the safety and efficacy for PRP in treating KIC in the real world.

## 4. Materials and Methods

### 4.1. Animals and Ketamine Administration

Thirty six female Sprague Dawley (SD) rats (Animal Center of BioLASCO, Taipei, Taiwan) weighed between 200 and 250 gm were randomly divided into four groups: (a) the control group (0.9% normal saline/500–700 μL/day, intraperitoneal, 8 weeks), (b) the ketamine group (Ketalar, Pfizer, 35 mg/kg/day, intraperitoneal, 8 weeks), (c) the ketamine + PRP group, with ketamine injection for 8 weeks and then intravesical instillation with PRP for 4 weeks (once a week), and (d) the ketamine + platelet-poor plasma (PPP) group, with ketamine injection for 8 weeks and then intravesical instillation with PPP for 4 weeks. Rats were weighed at the beginning of each week to adjust the amount of ketamine administered. This study was approved by the Animal Care and Treatment Committee of Kaohsiung Medical University (IACUC: KMUH-106086). All animals were maintained in individual cages at 22–25 °C, 12 h light/dark cycles, and given free access to food and water during all the experiments. All experiments were performed in accordance with laboratory animal care guidelines to minimize animal stress/distress.

### 4.2. Platelet-Rich Plasma (PRP) and Platelet-Poor Plasma (PPP) Supernatant Preparation

**Figure 8:**
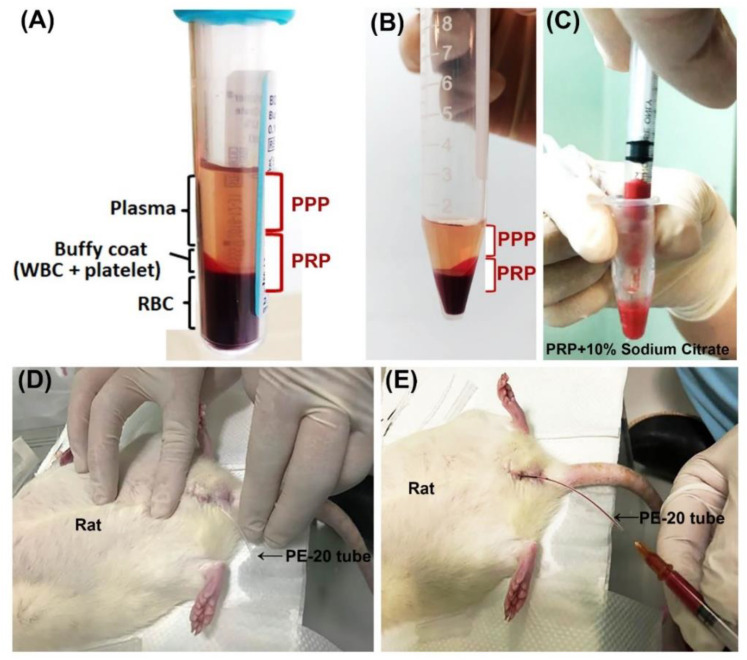
Schematization of platelet-rich plasma (PRP) preparation and application in rat. Blood was harvested from female rats through tail vein under anesthesia. (**A**) After centrifugation, the collected whole blood was separated into three layers, plasma layer, platelets, leukocytes and red blood cells from top to bottom, respectively. (**B**) After transferred the upper fraction to other new 5 mL vacuum tube and then centrifugation, the upper fraction was separated into platelet-poor plasma (PPP, upper fraction) layer and platelet-rich plasma (PRP, lower fraction). After mixed with 0.1 mL of 10% calcium chloride solution and 0.2 mL of PRP or PPP (**C**), the bladder of the animal was emptied (**D**) and 0.3 of the mixture (calcium chloride solution and PRP or PPP) was injected into bladder by PE-20 tube once per week (**E**).

4 mL of SD rat blood was drawn from four healthy donors through tail vein under anesthesia. The rat blood was collected by using a 5 mL disposable syringe containing 0.35 mL of 3.8% sodium citrate 3.15 mL of each animal blood with ratio of 9:1 was collected. Through centrifugation at 160 G for 20 min at 22 °C, whole blood was separated into three layers, plasma layer, platelets, leukocytes and red blood cells from top to bottom respectively. Then, the red cell component (lower red fraction) and serum component (an upper straw-yellow turbid fraction) were observed (Figure 8A). The upper fraction was carefully pipetted and transferred to other new 5 mL vacuum tube. The sample was then submitted to a new centrifugation at 400 G, for 15 min at 4 °C, resulting in two components: the upper fraction on the tube is known as platelet-poor plasma (PPP) and other lower fraction known as platelet-rich plasma (PRP) (Figure 8B). Similar amounts of PRP and PPP (0.2 mL) were transferred to different sterile tubes and then activated by 0.1 mL of 10% calcium chloride solution (ScienceLab.com Inc., Houston, TX, EUA) (Figure 8C). PPP contained smaller quantities of growth factors compared to PRP. The animals’ bladders were emptied prior to intravesical instillation of PRP or PPP (Figure 8D). 0.3 mL mixed with 0.1 mL of 10% calcium chloride solution and 0.2 mL of PRP or PPP into bladder by PE-20 tube once per week. (Figure 8E). Following, the catheters were removed after 30 min to ensure that they did not void the subsequently instilled PRP.

### 4.3. Cystometrogram and Data Analysis

To confirm whether ketamine-induced voiding dysfunction altered bladder function, cystometrograms (CMGs) studies were performed during ketamine infusion into bladder of anesthetized rat. According to the previously studies [8,88], rats were anesthetized with Zoletil-50 (1 mg/kg, intraperitoneal injection) and then the bladder was emptied before starting each CMG. After placing the indwelling urethral catheter (PE50 tube), saline was injected into the bladder at a steady rate (0.08 mL/min) and the pressure in the catheter was measured. After initial stabilization, CMG was recorded at least 5 filling/voiding cycles by a data acquisition system (ML866 PowerLab, ADInstrument, Colorado Springs, CO, USA) and digitized for computer data collection (Labchart 7, ADInstruments: Windows 7 system). Subsequently, the filling pressure, peak micturition pressure, bladder capacity, and frequency of non-voiding contractions (without urine leakage during bladder infusion) were analyzed. Bladder capacity was measured by the amount of saline that infused into the bladder at the commencement of micturition.

### 4.4. Physiological Metabolic Cage for Micturition Frequency/Volume Studies

Voiding behavior was recorded using metabolic cages after administration of ketamine. As previously studies described, injected rats were placed in individual R-2100 metabolic cages (Lab Products, Rockville, MD, USA). The previous conditions were maintained for a 24-h familiarization period. After this, a known volume of water was measured and placed in the animals’ drinking bottles. The 24-h micturition frequency and the volume of urine output were determined using a cup especially fitted to the MLT0380 transducer (ADInstruments, Colorado Springs, CO, USA) and recorded by a transducer (MLT 0380, ADI Instruments, Colorado Springs, CO, USA). The volume of water intake and urine output were also analyzed [8,60].

### 4.5. Bladder Contractility Studies

Based on previous described [60,89,90,91], the bladders were recorded for weight and cut into three longitudinal strips measuring 0.5 × 1.5 cm^2^ from the bladder dome to the trigone area. Contractile responses were performed using a force-displacement transducer. 2 g tissue was applied under a resting tension for 30 min. At this length, the maximum tension responses to electrical-field stimulation (2, 8, and 32 Hz), carbachol (20 μM) and KCl (120 mM) were recorded. The data were then digitized and analyzed using the Grass POLYVIEW A-D & conversion system (Grass Instrument Co., Warwick, RI, USA).

### 4.6. Ketamine Metabolites Assay in Urine and Serum

According to ISO 9001:2000 requirements and previously study [92], one ml of blood was obtained from the tail of rats and the serum was separated by centrifugation at 4 °C. The 24-h urine was collected by the metabolic cage. Urine and serum concentrations of ketamine and norketamine were determined using HPLC. After purification by liquid–liquid extraction with ethyl ether, urine samples were chromatographed on a reversed-phase column. The concentrations of ketamine and norketamine were then detected by UV spectrophotometry at 200 nm.

### 4.7. Histopathology

The current study was conducted to investigate pathological changes with Masson’s trichrome stain (Masson’s trichrome Stain Kit HT15, Sigma, St. Louis, MO, USA) and immunofluorescence staining following the manufacturer’s protocol and previously studies [8,60,93,94]. Histological analysis was examined and evaluated by two independent pathologists. Bladder tissue samples were fixed overnight in 4% paraformaldehyde solution, then embedded in paraffin, and sectioned into 5-μm thick sections. After deparaffinization and hydration, sections were stained using the Masson’s trichrome stain to detect the connective tissue (blue) and detrusor smooth muscle (DSM; red) in the current study.

For doubly immunofluorescence staining, whole bladder mounts were fixed overnight in cold 4% paraformaldehyde in PBS. After blocking with 10% NGS/0.5% Triton X-100 in PBS for 1 h at RT, the whole bladder mounts were incubated with primary at 4 °C overnight, followed by secondary antibodies (1:800; Invitrogen, Waltham, MA, USA) for 2 h at RT. The primary antibody were E-Cadherin (Proteintech, Rosemont, IL, USA, rabbit polyclonal IgG 1:100, catalog no. 20874-1-AP), Ki67 (Abcam, Cambridge, UK, rabbit monoclonal IgG 1:100, catalog no. ab1667), CK14 (Abcam, mouse monoclonal IgG, 1:250, catalog no. ab7800), Claudin-4 (Invitrogen, mouse monoclonal IgG, 1:100, catalog no. 329400), Neurofilament (BioLegend, San Diego, CA, USA, mouse monoclonal IgG, 1:100, MW: 200~220 kDa, catalog no. 837904), M2 (Eptomics, Burlingame, CA, USA, rabbit polyclonal IgG, 1:100, catalog no.3191-S), and alpha-smooth muscle actin (α-SMA; Abcam, rabbit polyclonal IgG, 1:100, catalog no. ab5694). Following, the sections were stained with DAPI and then coverslipped in Prolong Gold anti-fade reagent (Invitrogen). Each section was observed under a Zeiss LSM 700 confocal microscope (Carl Zeiss Ltd., Jena, Germany).

### 4.8. Western Blotting Analysis

The bladder mucosa was gently scraped from the muscle tissue following digestion using a forceps. Frozen bladder tissue samples were homogenized on ice in lysis buffer (50 mM Tris, pH 7.5, 5% Triton-X100) with Halt Protease Inhibitor Cocktail (Pierce, Rockford, IL, USA). Equal amounts of total protein (30 μg) from the bladders were loaded on 12% SDS polyacrylamide (SDS-PAGE) gels and transferred to Immobilon-P membranes. Immobilon-P membranes were then blocked with 5% non-fat milk in 0.05% Tween 20 in Tris-buffered saline at room temperature for 1 h and were then incubated with the primary antibody. Other materials and procedures used in Western blotting analysis experiments were described in Appendix A.

### 4.9. Statistical Analysis

All results are expressed as the mean ± standard deviation (SD) and were statistically analyzed by two-way analysis of variance, followed by Bonferroni test for individual comparison. Student’s *t*-test was used to calculate *p*-values for comparison. The *p*-values were calculated on triplicate experiments. For all tests, a *p*-value < 0.05 was set as statistically significant.

## 5. Conclusions

The present studies revealed that the effect of PRP regulated inflammatory fibrotic biosynthesis, attenuated oxidation, promoted mucosal regeneration, and enhanced angiogenesis and neurogenesis to improve bladder function and ameliorate the overactive bladder symptoms in rats with KIC. PRP could promote angiogenic potential for bladder repair through laminin/integrin-α6 and VEGF/VEGF-R signaling pathways in the pathogenesis of KIC. These findings suggested that the PRP therapy may offer new strategies for effective therapy in KIC.

## Figures and Tables

**Table 1:** Physical indicator, serum, urine and urodynamic for the different experimental group.

	Control	Ketamine	Ketamine + PRP	Ketamine + PPP
No. rats	10	10	8	8
Physical indicators				
Body weight (gm)	306.1 ± 19.0	278.9 ± 18.2	287.0 ± 16.5	292.7 ± 11.3
Bladder weight (mg)	138.0 ± 30.5	186.6 ± 41.0 **	140.8 ± 26.7 ^††^	168.9 ± 31.3 *^,†,^^#^
The ratio of bladder weight (mg)/body weight (gm)	0.45 ± 0.10	0.67 ± 0.16 **	0.48 ± 0.09 ^†^	0.58 ± 0. 12 *^,†,^^#^
Water intake (mL/24 h)	35.6 ± 3.4	36.5 ± 7.3	46.0 ± 7.5	47.3 ± 8.6
Urine output (mL/24 h)	18.2 ± 4.0	18.1 ± 4.6	16.5 ± 3.9	17.4 ± 4.2
Serum parameters				
Ketamine (ng/mL)	ND	ND	ND	ND
Norketamine (ng/mL)	ND	ND	ND	ND
Urine parameters				
Ketamine conc. (ng/mL)	ND	1018.6 ± 220.5 **	936.7 ± 197.8 **	976.3 ± 185.9 **
Norketamine conc. (ng/mL)	ND	9850.5 ± 946.8 **	8190.0 ± 835.4 **	8740.0 ± 933.7 **
Urodynamic parameters				
Frequency (No. voids/h)	4.63 ± 0.92	6.75 ± 2.76 *	4.13 ± 1.2 ^†^	4.29 ± 1.2 ^†^
Peak micturition pressure (cmH_2_O)	30.7 ± 4.1	41.56 ± 4.2 *	29.4 ± 3.0 ^†^	29.8 ± 2.6 ^†^
Voided volume (mL)	2.1 ± 0.3	1.5 ± 0.2 **	2.8 ± 0.4 *^,††^	2.4 ± 0.3 ^†^
No. non-voiding contractions between micturition (No./h)	0	3.2 ± 0.7 **	0	0
Interval (min)	13.0 ± 2.60	9.6 ± 4.4 **	15.6 ± 4.4 ^†^	14.3 ± 3.7 ^†^

Footnote: PRP, platelet rich plasma; PPP, platelet poor plasma; h, hour. Values are means ± SD. * *p* < 0.05; ** *p* < 0.01 versus control group; ^†^ *p* < 0.05; ^††^ *p* < 0.01 versus the ketamine group. ^#^ *p* < 0.05 versus the ketamine + PRP group.

## Data Availability

The datasets of the present study can be available from the corresponding author upon request.

## Appendix A. Other Materials and Procedures Used in Western Blotting Analysis Experiments

Membranes were incubated with the indicated primary antibodies for Uroplakin III (UPKIII; Abcam, mouse monoclonal IgG1, 1:1000, MW: 32 kDa, catalog no. ab78196), E-Cadherin (Proteintech, rabbit polyclonal IgG 1:1000, MW: 97 kDa, catalog no. 20874-1-AP), TGF-β1 (R&D, mouse monoclonal IgG 1:1000, MW: 25 kDa, catalog no. MAB240), fibronectin (Merck, mouse monoclonal IgG1, 1:1000, MW: 262 kDa, catalog no. MAB 1940), type 1 collagen (Abcam, rabbit polyclonal IgG, 1:1000, MW: 139 kDa, catalog no. ab34710), COX-2 (Cayman, rabbit polyclonal IgG, 1:1000, MW: ~74 kDa, catalog no. 160126), NF-κB P65 (Proteintech, rabbit polyclonal IgG, 1:1000, MW: 65 kDa, catalog no. 10745-1-AP), Ki67 (Abcam, rabbit monoclonal IgG 1:1000, MW: 358 kDa, catalog no. ab1667), CK14 (Abcam, mouse monoclonal IgG, 1:1000, MW: 55 kDa, catalog no. ab7800), CD44 (hyaluronic acid [HA] receptor) (Cell Signaling, mousemonoclonalIgG1,1:1000, MW: 80 kDa, catalog no. #5640), Claudin-4 (Invitrogen, mouse monoclonal IgG, 1:1000, MW 22 kDa, catalog no. 329400), Zonula occludens (ZO)-1 (ZO-1; Invitrogen, rabbit polyclonal IgG, 1:1000, MW 220 kDa, catalog no. 40-2200), Laminin (Abcam, rabbit polyclonal IgG, 1:1000, MW: 200~400 kDa, catalog no. ab7463), integrin-α6 (Laminin receptor) (Abcam, rabbit monoclonal IgG, 1:5000, MW: ~127 kDa, catalog no. ab181551), alpha-smooth muscle actin (α-SMA; Abcam, rabbit polyclonal IgG, 1:6000, MW: 40, 42 kDa, catalog no. ab5694), vascular endothelial growth factor (VEGF; Merck, mouse monoclonal IgG, 1:1000, MW: ~40 kDa, catalog no. MABC595), VEGF-R1 (VEGF receptor; Abcam, rabbit monoclonal IgG, 1:1000, MW: ~150 kDa, catalog no. ab32152), VEGF-R2 (VEGF receptor; Cell Signaling, rabbit monoclonal IgG, 1:1000, MW: ~220 kDa, catalog no. #9698), Neurofilament (BioLegend, mouse monoclonal IgG, 1:1000, MW: 200~220 kDa, catalog no. 837904), M2 (Eptomics, rabbit polyclonal IgG, 1:1000, MW: 52 kDa, catalog no.3191-S), M3 (Merck, rabbit polyclonal IgG, 1:1000, MW: 85 kDa, catalog no. ab87199), P2X3 (Abcam, rabbit polyclonal IgG, 1:1000, MW: 68 kDa, catalog no. ab109054), DNP (BETHYL, goat polyclonal IgG, 1:1000, MW: 40~50 kDa, catalog no. A150-117A), Nitrotyrosine (Merk, mouse monoclonal IgG, 1:1000, MW: 40~50 kDa, catalog no. MAB5404), manganese-containing superoxide dismutase (MnSOD; Abcam, rabbit monoclonal IgG, 1:1000, MW: ~25 kDa, catalog no. ab68155), copper/zinc superoxide dismutases (Cu/ZnSOD; Abcam, rabbit monoclonal IgG, 1:1000, MW: 16~18 kDa, catalog no. ab51254), catalase (Abcam, rabbit polyclonal IgG, 1:1000, MW: 60 kDa, catalog no. ab16731), and glyceraldehyde-3-phosphate dehydrogenase (GAPDH; Merck, mouse monoclonal IgG, 1:2500, MW: 36 kDa, catalog no. MAB374). Immunoreactive bands were visualized by enhanced chemiluminescence (ECL) and exposed to Biomax L film (Kodak). In each experiment, negative controls were also done without primary antibody.

Inflammatory fibrosis proteins (TGF-β1, fibronectin, type 1 collagen and COX-2), cell proliferation and differentiation proteins (Ki67, CD44, CK14 and UPKIII), urothelial tight junction-associated proteins and adhesion molecule (Claudin-4, ZO-1 and E-Cadherin), angiogenesis related proteins and receptors (laminin, integrin-α6, α-SMA, CD31, VEGF, VEGF-R1, VEGF-R2), neurogenesis related proteins and receptors (Neurofilament, M2, M3 and P2X3), oxidative stress markers (DNP and nitrotyrosine) and antioxidant enzymes (MnSOD, Cu/ZnSOD and catalase were normalized with ß-actin (Millpore, mouse monoclonal IgG1, 1:1000) and GAPDH.
