# Correction: Chueh et al. Therapeutic Effect of Platelet-Rich Plasma Improves Bladder Overactivity in the Pathogenesis of Ketamine-Induced Ulcerative Cystitis in a Rat Model. *Int. J. Mol. Sci.* 2022, *23*, 5771

**DOI:** 10.3390/ijms27073116

**Published:** 2026-03-30

**Authors:** Kuang-Shun Chueh, Kuan-Hua Huang, Jian-He Lu, Tai-Jui Juan, Shu-Mien Chuang, Rong-Jyh Lin, Yi-Chen Lee, Cheng-Yu Long, Mei-Chen Shen, Ting-Wei Sun, Yung-Shun Juan

**Affiliations:** 1 Graduate Institute of Clinical Medicine, College of Medicine, Kaohsiung Medical University, Kaohsiung 80708, Taiwan; spacejason69@yahoo.com.tw (K.-S.C.); urolong@yahoo.com.tw (C.-Y.L.); 2 Department of Urology, Kaohsiung Municipal Ta-Tung Hospital, Kaohsiung 80145, Taiwan; 3 Department of Urology, Kaohsiung Medical University Hospital, Kaohsiung 80708, Taiwan; u9181002@gmail.com (S.-M.C.); bear5824@gmail.com (M.-C.S.); selina750220@yahoo.com.tw (T.-W.S.); 4 Divisions of Urological Oncology, Department of Surgery, Chi Mei Medical Center, Tainan 71004, Taiwan; skhsteven@gmail.com; 5 Emerging Compounds Research Center, Department of Environmental Science and Engineering, College of Engineering, National Pingtung University of Science and Technology, Pingtung 91201, Taiwan; toddherpuma@yahoo.com.tw; 6 Department of Medicine, National Defense Medical College, Taipei 11490, Taiwan; terry870921@gmail.com; 7 Department of Parasitology, School of Medicine, College of Medicine, Kaohsiung Medical University, Kaohsiung 80708, Taiwan; rjlin@kmu.edu.tw; 8 Graduate Institute of Medicine, College of Medicine, Kaohsiung Medical University, Kaohsiung 80708, Taiwan; 9 Department of Medical Research, Kaohsiung Medical University Hospital, Kaohsiung 80708, Taiwan; 10 Department of Anatomy, School of Medicine, College of Medicine, Kaohsiung Medical University, Kaohsiung 80708, Taiwan; yichen83@kmu.edu.tw; 11 Department of Obstetrics and Gynecology, Kaohsiung Medical University Hospital, Kaohsiung 80708, Taiwan; 12 Department of Obstetrics and Gynecology, Kaohsiung Municipal Hsiao-Kang Hospital, Kaohsiung 80708, Taiwan; 13 Regenerative Medicine and Cell Therapy Research Center, Kaohsiung Medical University, Kaohsiung 80708, Taiwan

## Error in Figures

In the original publication [1], there was a mistake in Figure 1A (Control group, the Ketamine group, and the Ketamine + PRP group), Figure 2G (the Ketamine + PRP group), Figure 2H (the Ketamine + PPP group) and Figure 3G (the Ketamine + PRP group) as published. Image panels were mistakenly duplicated or misplaced during figure preparation. The corrected Figure 1, Figure 2 and Figure 3 appear below. The authors state that the scientific conclusions are unaffected. This correction was approved by the Academic Editor. The original publication has also been updated.

## Figures and Tables

**Figure 1:**
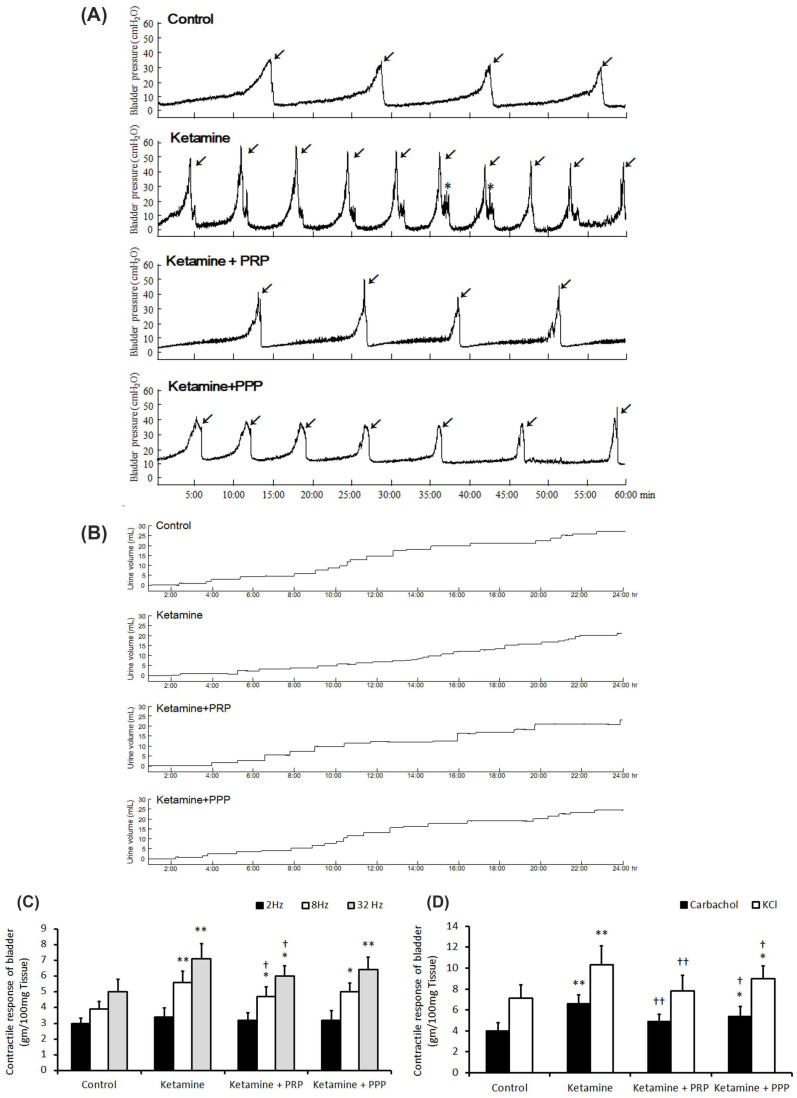
Cystometric parameter, voiding behavior and contractile responses. (**A**) Cystometric recordings performed for micturition pressure, voiding volume, and urinary frequency, including voiding contraction (arrows) and non-voiding contraction (asterisks). (**B**) Tracing analysis of voiding behavior for 24 h by metabolic cage. The ketamine group exhibited significantly increases in bladder micturition pressure, voiding contractions, non-voiding contractions and micturition frequency, whereas PRP and PPP treatment meaningfully ameliorated bladder micturition pattern and capacity. (**C**,**D**) Contractile responses of bladder strips and statistics data were examined by electrical field stimulation (2 Hz, 8 Hz and 32 Hz) (**C**), carbachol and KCl (**D**). Data were expressed as means ± SD for *n* = 4, * *p* < 0.05; ** *p* < 0.01 versus the control group; ^†^ *p* < 0.05; ^††^ *p* < 0.01 versus the ketamine group.

**Figure 2:**
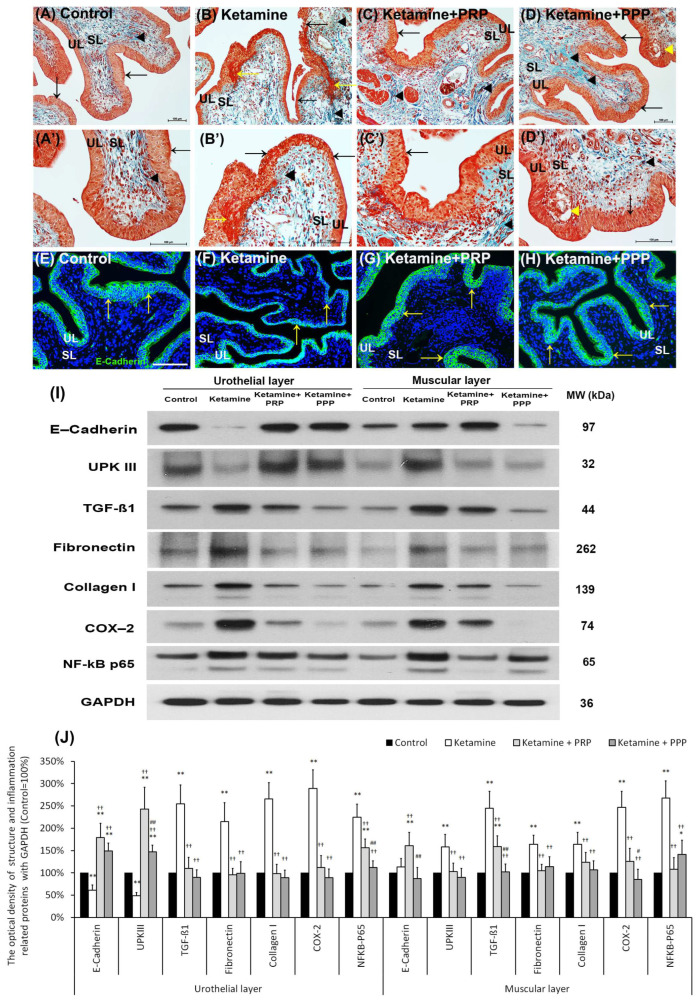
Therapeutic effect of PRP improved ketamine-induced pathological alteration by Masson’s trichrome staining, immunofluorescence and Western blotting analysis. (**A**–**D**’) Bladder pathological features of the control group (**A**,**A’**), the ketamine group (**B**,**B’**), the ketamine + PRP group (**C**,**C’**) and the ketamine + PPP group (**D**,**D’**). Masson’s trichrome stain showed red-stained smooth muscle, navy blue-stained nucleus, green-stained collagen. In the control group (**A**,**A’**), there were three to five layers of the urothelial layer (UL) (black arrow) and only scattered collagen (black arrowhead) distributed in the suburothelial layer (SL; lamina propria). In the ketamine group (**B**,**B’**), the morphology was characterized by thinner layer of urothelial cells (black arrows), hemorrhage (yellow arrows), much collagen accumulation (black arrowheads) and increased bladder fibrosis. In contrast, the pathological features of the ketamine + PRP group (**C**,**C’**) and the ketamine + PPP group (**D**,**D’**) showed recovered ketamine-induced bladder damages by increasing thicker layer of urothelium (black arrows) and reducing interstitial fibrosis (black arrowheads) when comparing to the ketamine group. Especially, there was a vacuolation beneath urothelial layer (yellow arrowheads) in the ketamine + PPP group (**D**,**D’**). (**E**–**H**) The expression of cell-adhesion protein E-cadherin by immunostaining was shown. In the control group (**E**), the E-cadherin staining was found in urothelial intercellular junctions. In contrast, there was less E-cadherin staining distribution in thin urothelium of the ketamine group (**F**), but the immunostaining of the ketamine+ PRP group (**G**) and the ketamine+ PPP group (**H**) were enhanced in urothelium. (**I**,**J**) Western blotting analysis was performed to evaluate the protein levels of urothelial structure (E-cadherin and UPKIII) and bladder inflammation (TGF-ß1, COX-2 and NF-κB), interstitial fibrosis (fibronectin and type I collagen). Both the inflammatory and fibrosis markers (TGF-ß1, fibronectin, type I collagen, COX-2 and NF-κB) were meaningfully elevated in UL and ML of the ketamine group when comparing to the control group. Moreover, the proteins were markedly decreased in the ketamine + PRP group and the ketamine + PPP group when comparing to the ketamine group. Results were normalized as the control = 100%. UPKIII, uroplakin III; TGF-ß1, transforming growth factor ß1. Data were expressed as means ± SD for *n* = 6, * *p* < 0.05; ** *p* < 0.01 versus the control group; ^††^ *p* < 0.01 versus the ketamine group; ^#^ *p* < 0.05; ^##^ *p* < 0.01 versus the ketamine + PRP group.

**Figure 3:**
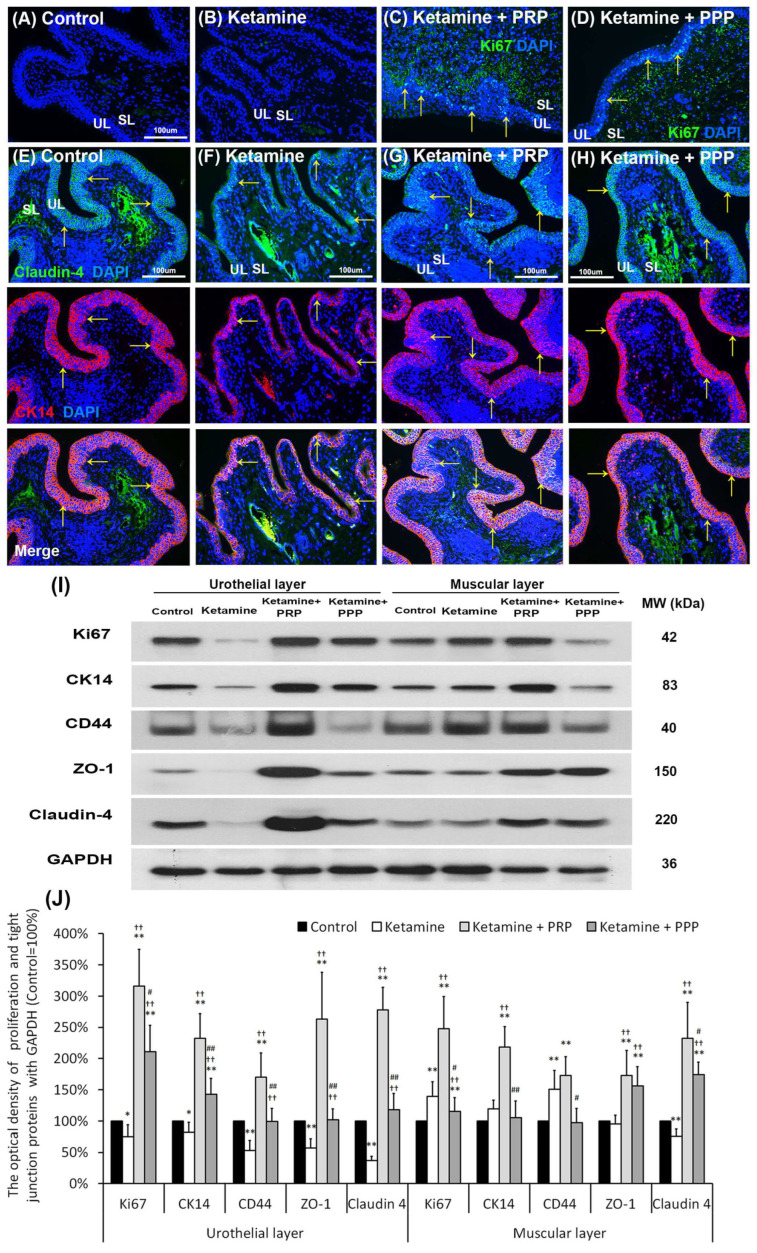
PRP strengthened urothelial proliferation and junction-associated protein expression. The expressions of proliferating and differential marker (Ki67, CK14 and CD44) markers and urothelial tight junction (Claudin-4 and ZO-1) were assessed by immunofluorescence evaluation (**A**–**H**) and Western blotting analysis (**I**,**J**). (**A**–**D**) The staining of Ki67 (a proliferation marker) was less expression in the bladder tissues of the control group (**A**), and the ketamine group (**B**). On the contrary, the Ki67 immunostaining was expressed in the urothelial basal layer in the ketamine + PRP group (**C**) and the ketamine + PRP group (**D**). (**E**–**H**) The co-staining of claudin-4 and CK14 (yellow arrows) was widely expressed in the urothelial layer. Double-labeled staining of Claudin-4 (fluorescein isothiocyanate; green, **upper panels**) and CK14 (rhodamine; red, **lower panels**) (yellow arrows) was widely expressed in the urothelial layer in the control group (**E**). The co-staining was restricted to the thin and disrupted urothelium in ketamine group (**F**). Conversely, the staining of the ketamine + PRP group (**G**) and the ketamine + PPP group (**H**) was markedly distributed in the urothelial basal layer compared to the ketamine group (**F**). Nuclear DNA was labeled with DAPI (blue). (**I**,**J**) Western blotting analysis was used to quantified the percentage of Ki67, CK14, CD44, Claudin-4 and ZO-1. The protein levels were meaningfully enhanced in the ketamine + PRP group when comparing to the ketamine group. Results were normalized as the control = 100%. CK, cytokeratin. Data were expressed as means ± SD for *n* = 6, * *p* < 0.05; ** *p* < 0.01 versus the control group; ^††^ *p* < 0.01 versus the ketamine group; ^#^ *p* < 0.05; ^##^ *p* < 0.01 versus the ketamine + PRP group.
